# Conductive Atomic Force Microscopy—Ultralow-Current Measurement Systems for Nanoscale Imaging of a Surface’s Electrical Properties

**DOI:** 10.3390/s24175649

**Published:** 2024-08-30

**Authors:** Andrzej Sikora, Krzysztof Gajewski, Dominik Badura, Bartosz Pruchnik, Tomasz Piasecki, Kamil Raczkowski, Teodor Gotszalk

**Affiliations:** 1 Department of Nanometrology, Faculty of Electronics, Photonics and Microsystems, Wrocław University of Science and Technology, 50-370 Wrocław, Poland; k.gajewski@gmail.com (K.G.); dominik.badura@pwr.edu.pl (D.B.); bartosz.pruchnik@pwr.edu.pl (B.P.); tomasz.piasecki@pwr.edu.pl (T.P.); kamil.raczkowski@pwr.edu.pl (K.R.); teodor.gotszalk@pwr.edu.pl (T.G.); 2 Nokia Solutions and Networks, ul. Lotnicza 12, 54-155 Wroclaw, Poland

**Keywords:** atomic force microscopy, electrical properties, nanoscale resistance mapping, ultralow-current measurements, conductive atomic force microscopy, scanning spreading resistance microscopy

## Abstract

One of the most advanced and versatile nanoscale diagnostic tools is atomic force microscopy. By enabling advanced imaging techniques, it allows us to determine various assets of a surface, including morphological, electrical, mechanical, magnetic, and thermal properties. Measuring local current flow is one of the very important methods of evaluation for, for instance, photovoltaic materials or semiconductor structures and other nanodevices. Due to contact areas, the current densities can easily reach above 1 kA/m^2^; therefore, special detection/measurement setups are required. They meet the required measurement range, sensitivity, noise level, and bandwidth at the measurement scale. Also, they prevent the sample from becoming damaged and prevent unwanted tip–sample issues. In this paper, we present three different nanoscale current measurement solutions, supported with test results, proving their performance.

## 1. Introduction

Electrical nanoscale characterization of materials and structures plays a crucial role in modern science, providing insights into the phenomena observed in semiconductor materials [1], two-dimensional materials [2], photovoltaic materials and modules [3,4], complex microelectronic devices [5,6] or even quantum states [7]. Within the vast range of measuring techniques available, where a conductive probe is utilized and the tip–sample current is determined in a quantitative fashion, continuous progress in terms of measuring tools themselves is a constant requirement [8,9,10]. Particularly essential are probes [11,12], as well as the methodology and protocols [13,14,15,16], including data interpretation [17,18]. In order to provide flexible, wide-range, high-quality current measurements, we developed and tested various solutions that can be utilized with commercial and home-made microscopes.

Mapping the electrical properties of a surface at the nanoscale is often performed using conductive atomic force microscopy (C-AFM) or scanning spreading resistance microscopy (SSRM) modes. These two modes show linear (C-AFM) or logarithmic (SSRM) input current transfer functions. C-AFM enables current sensing from tens of femtoamperes up to single milliamperes, but typically 3–4 orders of magnitude during a single measurement. Current measurement in the typical C-AFM mode is conducted with a linear transimpedance amplifier with optional additional output voltage amplification [19], which may contain several switchable feedback gains for variable amplification. Other variations may utilize differential input [20], a T-network in the feedback loop [21], and additional ac-boosting stages [22]. Different setups may exhibit higher cut-off frequencies, but at the cost of having a higher noise level or a nonlinear noise–frequency dependence [22]. It has to be emphasized that the main drawback of recently available solutions for C-AFM and SSRM imaging is that each technique requires different application modules to be installed and used. Necessary module exchange makes it difficult to image the same area of interest using both modes. This action is troublesome due to the risk of probe/sample damage and the additional time needed for tip-sample calibration and positioning.

To overcome the limitations of specific designs, we developed three circuitry solutions, enabling low-current measurements in advanced, conductive probe AFM techniques: a multiple feedback transimpedance amplifier, an integrating current-to-voltage converter (CVC) module, and an SSRM current-reference module. The design and working principles are presented here, as well as the test results. The presented modules were successfully integrated into commercial and home-made AFM systems.

## 2. Theory of Operation

### 2.1. Multiple Feedback Transimpedance Amplifier

In the case of SSRM, current sensing typically ranges from a few picoamperes up to single milliamperes. The novelty of the presented multiple feedback transimpedance amplifier (MFTIA) is the integration of C-AFM and SSRM modes in one device. This removes the necessity of the application modules to be able to change between C-AFM and SSRM measurements.

**Figure 1:**
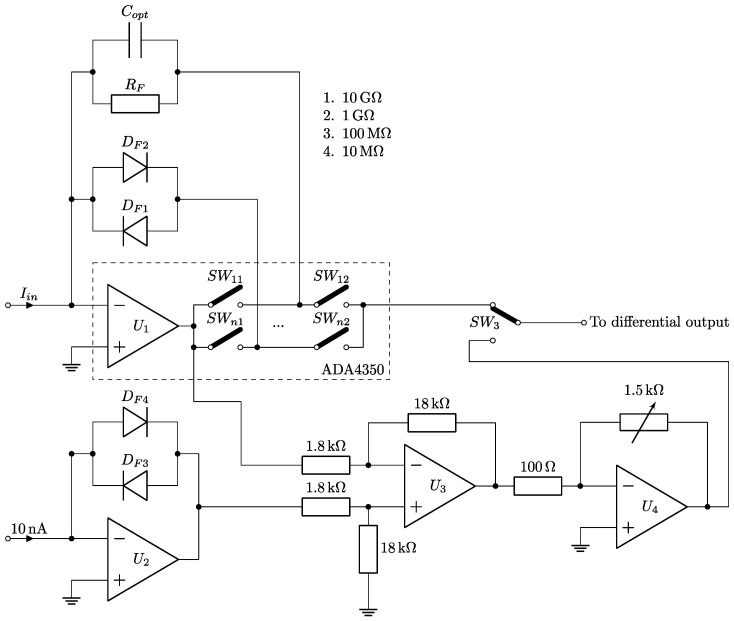
Simplified schematic of the MFTIA.

An MFTIA consists of the following elements: a transimpedance amplifier, a power supply unit, and a control unit (Figure 1).

In the transimpedance amplifier, the main element is an ADA4350–FET input analog front end with an ADC driver [23]. It contains an FET input operational amplifier, U_1_; a switching network, SW_nm_; and an ADC (analog/digital converter) driver. Its input bias current, ±0.25 pA (typical at 25 °C), is an ideal compromise for subpicoampere current sensing while maintaining a wide dynamic response for both the C-AFM and SSRM measurement modes, as low-input-bias-current operational amplifiers are usually slow. An integrated switching network consists of six independent sections. They can be controlled using a serial peripheral interface (SPI) or parallel control logic (in which case five sections are available). A switching network uses the Kelvin switching technique to decrease gain errors or offset errors which can occur when traditional analog switches are used. Additionally, the usage of an integrated switching network makes the MFTIA more compact, decreasing the occupied space. 

The MFTIA is equipped with four linear feedback resistors, R_F_, with the following resistances—10 MΩ, 100 MΩ, 1 GΩ, and 10 GΩ—enabling both conductive and tunneling current measurements. They were complemented with a pair of BAS116 diodes (D_F1_, D_F2_), enabling logarithmic current sensing in two directions. To fully realize SSRM, logarithmic detection was linearized with reference to a 10 nA current. For that purpose, a set of operational amplifiers, U_2_ − U_4_, with a pair of BAS116 diodes was added. The logarithmic current from ADA4350 was compared in U_3_ with the 10 nA reference, resulting in a 0 V output when a 10 nA current was measured. U_4_ amplifies the SSRM signal to obtain an amplification equal to 1 V/dec.

A leakage current of 3 pA expands the current sensing possibilities in comparison to other known constructions [24,25]. In order to minimize power supply noise, ultrahigh-power-supply rejection ratio linear regulators were chosen for positive and negative voltages (LT3045 and LT3094, Analog Devices, Wilmington, MA, USA). Their typical RMS noise was set at a level of 0.8 µV_RMS_ in the range of 10 Hz to 100 kHz. The linear regulators were set to ±6 V. An additional +3.3 V power supply line was prepared for the control unit. The control unit is responsible for setting the measurement mode and amplification. It is based on the STM32G071 microcontroller (STMicroelectronics, Geneva, Switzerland). The switching measurement mode was performed using an additional monolithic CMOS single-pole/double-throw switch (ADG1419, Analog Devices).

### 2.2. Integrating Current-to-Voltage Converter Module

**Figure 2:**
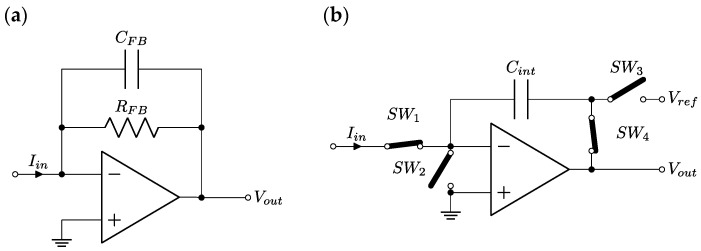
Simplified schematics of current-to-voltage converters: transimpedance amplifier (**a**) and integrating CVC based on part of the DCC112 integrated circuit (**b**).

Another method, concurrent to the traditional linear current-to-voltage converter (CVC) based on a transimpedance amplifier (Figure 2a), is the integrating CVC (Figure 2b). In a typical C-AFM application, the crucial parameter is the distance from the probe to the CVC, as per the line length noise and leakage increase. The dimensions of the CVC set are therefore limited by the measurement head. This is especially demanding for resistors, which tend to be larger with high resistance values while maintaining good quality. Their size and number, in case range switching is needed, also affect leakage and introduce capacitances, which then limit the bandwidth. Therefore, one of the biggest challenges is the high value of the feedback resistors, $R_{FB}$, many of which must be used together with a low-leakage multiplexer for range switching, as well as the bandwidth limitation resulting from parasitic or intentional capacitances, $C_{FB},$ parallel to $R_{FB},$ which are usually in the range of one to several picofarads.

The integrating CVC (Figure 2b) relies on charging or discharging the integrating capacitor, $C_{int},$ with the measured current, $I_{in},$ for a given integration time, $t_{int}$. The change in the output voltage of the integrating CVC will be $\Delta V_{out}=-\frac{I_{in}\;t_{int}}{C_{int}}$; therefore, the conversion ratio may be changed by modifying $t_{int},$ which is very easy to carry out digitally by using frequency dividers clocked from the reference clock signal.

The integrating CVC used in the C-AFM measurements was based on the Texas Instruments DDC112 integrated circuit [1]. The details of the integrating CVC and its performance were included in our previous work [2]. DDC112 is a two-channel integrating CVC accompanied by a 20-bit ΣΔ ADC. A single channel consists of two integrators working in tandem in two phases. The simplified schematic of one integrator is shown in Figure 2b. The shown switches’ positions are relevant to the integrating phase, in which $C_{int}$ is discharged from the $V_{ref}$ voltage with the input current starting at time t=0. At $t=t_{int}$ the input switch, $SW_{1},$ is opened, and the corresponding switch in the identical second integrator is closed, and it starts integrating. The output voltage is converted by the built-in ADC and then the switches $SW_{2}$, $SW_{3,}$ and $SW_{4}$ change positions, resulting in pre-charging of $C_{int}$ to the $V_{ref}$ voltage. At $t=2t_{int}$ the first integrator is ready and the measurement cycle is repeated. Of course, during the first half of the cycle, the ADC converts the output voltage from the second integrator. This results in the digitally converted data representing the measured currents with a sampling ratio of $\frac{1}{t_{int}}$ and a Nyquist-limited bandwidth of $\frac{1}{2t_{int}}$.

Additionally, what was not shown in simplified Figure 2b was that each CVC channel in DDC112 [26] allows for $C_{int}$ values to be varied in the range of 12.5 pF to 87.5 pF in 12.5 pF steps, which can also be achieved by using an externally connected capacitor. To improve the resolution and minimize the effect of the $V_{ref}$ voltage noise, the value of the $V_{ref}$ was stored in a sample-and-hold circuit, and it was subtracted from the $V_{out}$ before the ADC conversion [1].

### 2.3. SSRM Reference Current-Based Module

**Figure 3:**
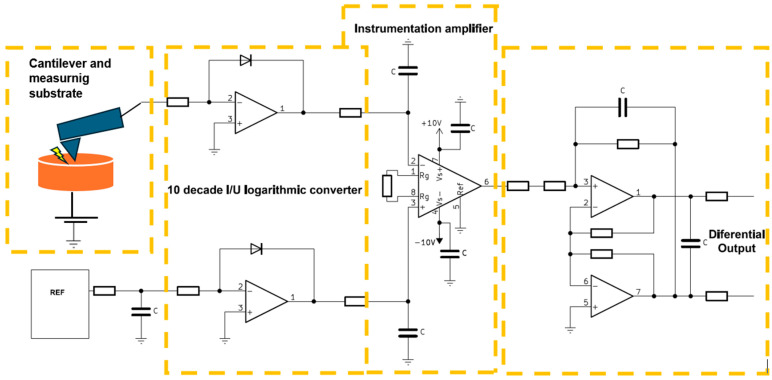
Simplified schematic of the SSRM I/U converter.

Conductive atomic force microscopy (C-AFM) is particularly useful for studying conductive materials such as semiconductors, metallic coatings, or nanoelectronic structures. Measuring this tip–sample current provides information about the local conductivity of the sample surface. The presented module consists of the following elements: transimpedance amplifiers (main and reference), an instrumentation amplifier, and a differential output (Figure 3).

**Figure 4:**
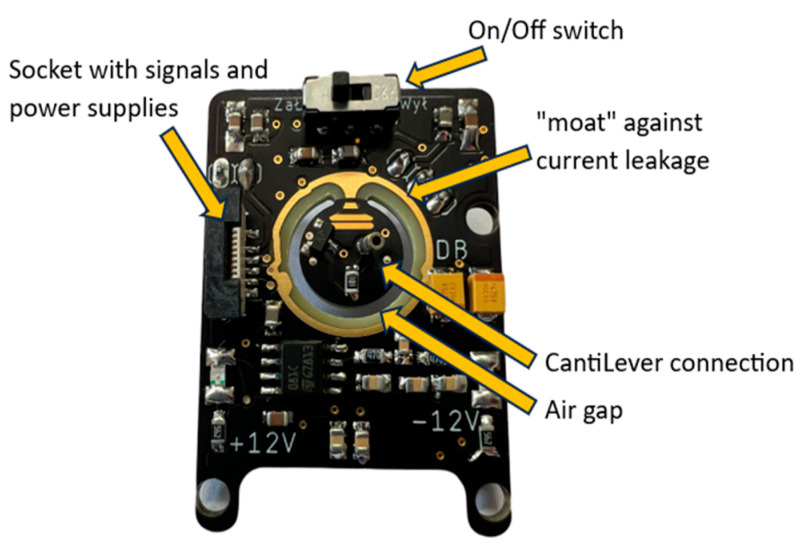
Developed logarithmic I/U converter.

The main component of the converter module is a precision JFET operational amplifier characterized by low power consumption and rail-to-rail output. The output of the circuit remains constant above a capacitive load of 500 pF. The design guidelines were that the circuit should have as broad a bandwidth as possible, a low offset, and a high-impedance input stage, preferably using n-channel JFET transistors. For the transimpedance amplifier, the AD8627 circuit was chosen to keep the input currents at the picoampere level over the entire input voltage range, which is extremely important when measuring in the C-AFM mode. The voltage noise remained at 16 nV/√Hz, which means it maintained a relatively low noise level at low frequencies, and its contribution to the signal noise when measuring high-resistance materials was negligible. In the feedback of the transimpedance amplifier is a BAV45 diode, which provides logarithmic characteristics of the I/U converter, which contributes to high dynamics during measurements. The presented circuit is less susceptible to noise and interference when measuring very small currents since logarithmization can naturally suppress the effect of noise relatively more effectively for small signals. The AD8221 instrumentation amplifier was used to provide a known reference point during the measurement by subtracting the logarithmically converted reference current from the measured one. The instrumentation amplifier was also configured to amplify the result of the input voltage by a factor of 10. The last part of the circuit is a dual operational amplifier that provides a symmetrical output for the circuit, which is then connected to an 18-bit ADC board. The constructed system is shown in Figure 4.

## 3. Material and Methods

The characterization of the SSRM circuit was carried out by measuring the current–voltage characteristics and performing C-AFM/SSRM measurements. The C-AFM/SSRM measurements were performed using a proprietary “Golden Piston” atomic force microscope. Conductively active piezoresistive probes by Nanoanalytik GmbH (Ilmenau, Germany), model RS3AP 1, platinum-coated tip of radius <10 nm, resonant frequency 50 kHz, spring constant 10 N/m) were used for scanning. The typical polarization voltage of the sample was 0.05 V–0.1 V, and the contact force was 30–50 nN. The imaged sample was graphite flakes (HOPG). The sample was attached to a holder that allowed for imaging the sample’s cross-section. To facilitate measurements at the edge, the sample was not positioned perpendicular to the scanning tip but at an angle of 27 degrees.

The verification of the designed MFTIA was carried out by characterization of the transfer function of the device and C-AFM/SSRM measurements. The I-V characteristic of each MFTIA mode was obtained using a Keithley 2602B source-measure unit and a Keithley 2001 multimeter. C-AFM/SSRM measurements were carried out using commercial AFM Veeco/Bruker Nanoman V equipped with a Nanoscope V controller. Arrow CONTPt (NanoWorld, Neuchâtel, Switzerland), platinum-coated, spring constant 0.2 N/m, resonant frequency 14 kHz) probes were used for scanning. The typical sample bias was 0.1 V, and the contact force was 30 nN. The imaged sample had a CS04-SiB calibration structure (IMEC), containing areas with different doping levels. The sample was attached to the holder, enabling cross-section imaging of the sample. To facilitate measurements at the edge, the sample was not set perpendicular to the scanning tip but at an angle of 60 deg. Electric contact with the sample was achieved using silver conductive paint (Silver Conductive 60 Adhesive 503, Electron Microscopy Sciences, (Hatfield, PA, USA).

## 4. Test Results

In order to verify the performance of the developed solutions, both calibration and test sample measurements were performed. The acquired data allowed us to confirm that the expected parameters were obtained for the current sense modules.

### 4.1. MFTIA Module Tests

**Figure 5:**
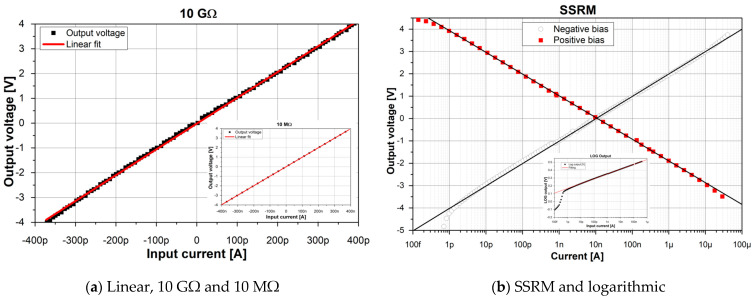
Result of calibration measurements of the MFTIA set to linear (**a**) and logarithmic (**b**) measurement modes. Feedback resistors were 10 GΩ and 10 MΩ (inset in (**a**)). Calibration curves for SSRM and logarithmic modes (inset (**b**)).

The results of the calibration measurements are presented for linear feedback (Figure 5a) as well as for logarithmic/SSRM feedback (Figure 5b). The obtained calibration curves are in agreement with the physical theory, according to which the output voltage, $U_{out},$ is either a linear function of the current signal, $I_{probe,}$ with a slope equal to the feedback resistance R,

(1) $$U_{out}=RI_{probe},$$

or a logarithmic function of the current signal, $I_{probe,}$ with reference to the reference current, $I_{ref},$ and slope obtained from the feedback diode characteristic and circuit amplification, *d*:

(2) $$U_{out}=\log_{10}\left(\frac{I_{probe}}{I_{ref}}\right)\cdot d$$

A special note has to be taken for the logarithmic/SSRM modes. The declared leakage current of BAS116 diodes is 3 pA. Its effect on the calibration curves can be observed in the inset of Figure 5b. 

For currents higher than 3 pA, the logarithmic dependence of the output voltage is clearly visible. Below 3 pA, the logarithmic curve starts to deviate, which can be a sign of leakage current in the diode. Note that positive-bias deviation from the logarithmic curve is observed for a current equal to 2–3 pA, which is in accordance with declared diode parameters. For negative bias, such deviation occurred at approximately 500 fA. This can be attributed to slightly different diode characteristics. An output voltage equal to 0 V is visible for 10 nA, which corresponds to the reference current (set by the 100 MΩ resistor).

**Figure 6:**
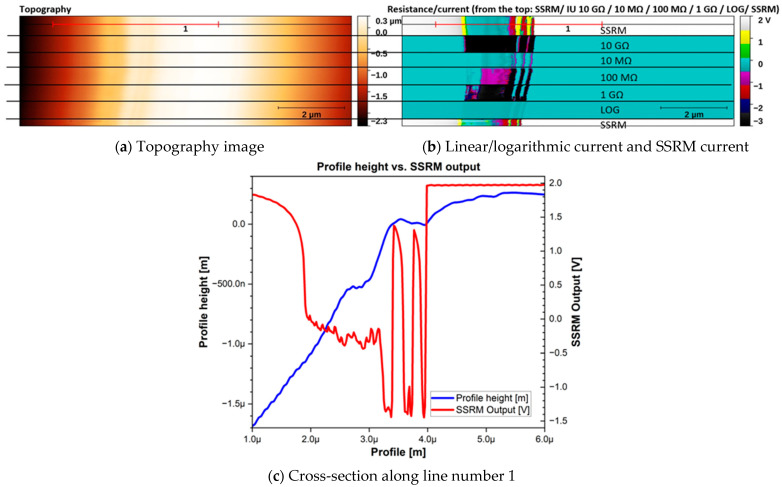
Results of CAFM/SSRM analysis of the SSRM calibration sample. Y scan axis was held so the same line was observed. Electrical mode/current sensitivity was switched during scanning. Observed sample resistivity varied from 2–5 MΩ (SSRM output equal~−1.4 V) to 10 GΩ (SSRM output equal~+2 V).

The utility of the designed MFTIA was verified using an SSRM calibration sample CS04-SIB, (IMEC, Leuven, Belgium). This sample contained stepwise layers of changing resistivity. On top of and on the substrate side of the sample, there were layers of high resistivity (Figure 6). The topography image (Figure 6a), current image (Figure 6b), and profile image, taken from the same row as the topography and current images (Figure 6c, selected fragment marked on red from Figure 6a,b), are presented. The input feedback of the MFTIA was changed between each mode (10 MΩ, 100 MΩ, 1 GΩ, 10 GΩ, LOG, and SSRM). The area of selected mode used was marked as black horizontal line on Figure 6a,b. Additionally, Figure 6b contains information, which mode was exactly used. 

An AFM scanner was held in the Y axis, so the same area of the sample was recorded. Any movement observed in the images came from thermal or mechanical drift of the scanning head (i.e., after switching the feedback of the MFTIA). The left side of the scanned area shows the top edge of the sample, whereas the right side of the image shows the substrate area of the sample. One can expect poor conductive properties in the area of the top edge of the sample, while in the substrate area, a very high resistance can be anticipated. In between, there is a visible area consisting of layers with different doping of the calibration sample. The resistivity of this area of the sample should be the lowest from the top side of the sample and the highest from the substrate side of the sample. This is consistent with the recorded current profile. In the case of the sample bias, which was positive in the presented case (+100 mV), the resultant recorded voltage for the SSRM mode should be the lowest for the lowest resistivity and becomes higher as the resistivity increases. The nonstepwise curve doping visible on the profile between 3.2 and 4 µm may arise from the existence of residues on the sample or scratch preparation before measurements, but this does not affect the testing possibilities of the device. The shorter length of the recorded active area comes from the way the sample was mounted for scanning.

Due to the bias direction and the output polarity in the case of the linear feedback mode, low conductivity results in outputs close to 0 V, and this decreases to negative values if the conductivity increases. It is apparent that no substantial tip movement arose as a result of changing the input feedback. This is contrary to the case when SSRM and C-AFM application modules are used separately (in which case stopping the measurement, exchanging application modules, and repeating the measurement protocol should be carried out). Additionally, when resistive feedback was changed, slightly different areas of the sample were properly mapped in terms of local current flow. When the feedback was set to 10 MΩ, all the scanned areas could be mapped. A comparison between the results obtained with various feedback resistances clearly shows obvious benefits for simple control over the CVC gain, which may be adjusted to image the less conductive areas in enough detail without data loss in highly conductive areas which saturate the CVC.

### 4.2. Integrating Current-to-Voltage Converter Module Tests

**Figure 7:**
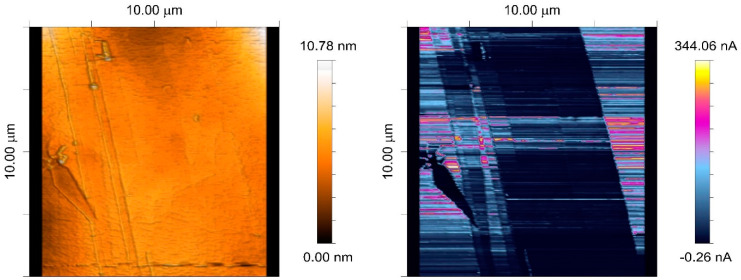
Topography (**left**) and current (**right**) scan of size 10 × 10 μm of the HOPG surface at 50 mV bias.

The integrating CVC was characterized to determine its performance and resolution. It achieves an input noise density ranging from 67.0 fA/√Hz for a 1 kHz bandwidth and 688.12 nA full-scale range down to 2.3 fA/√Hz for a 10 Hz bandwidth and 980 pA full-scale range [27]. Depending on the range, the RMS value of the noise was between 3.1 and 7.5 ppm of the full-scale current [2], and it rose to 5.71 and 8.21 when the input capacitance was 220 and 470 pF, respectively. The integrating CVC was used in the C-AFM measurement of the HOPG sample, as shown in Figure 7.

### 4.3. SSRM Reference Current-Based Module Tests

**Figure 8:**
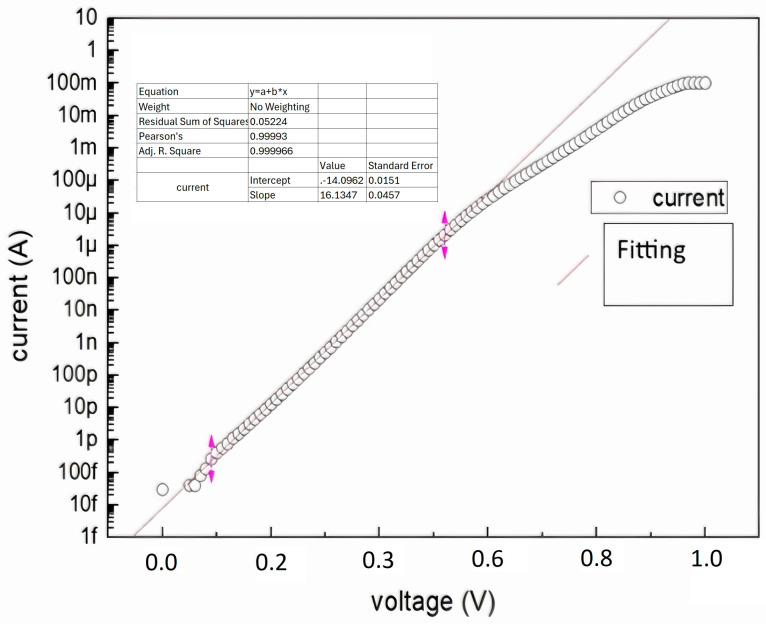
Current–voltage characteristics of the converter. The arrows show data range used for fitting.

The I/U characteristics of the SSRM module were measured using a Keithley 2634A SMU and are presented in Figure 8. Logarithmic conversion was obtained at currents ranging from 100 fA to 10 µA, while measurements were performed for currents ranging from 10 fA to 10 mA. Below 100 fA, the measured current value is interfered with by leakages present in the circuit; above 10 μA, linearity is disrupted by the serial resistance of the diode. Due to the use of a single diode, only unipolar values of current are measurable. Calibration performed using the Keithley 2634B SMU allowed us to establish the relation between the output voltage and the probe current:

(3) $$I_{Probe}=10^{\left(-15.36\times U_{out}-15.13\right)}[A]$$

Knowing the value of the substrate’s U_bias_ polarization voltage and the value of the I_probe_ current measured by the lever, the point resistance of the measured substrate can be determined using Ohm’s transformed law:

(4) $$R=\frac{U_{bias}}{I_{probe}}[\Omega ]$$

Calibration shows that SSRM is a useful tool for current measurements within eight orders of magnitude. Therefore, it is a perfect tool for measurements of the resistance of technical materials with large resistivity variations or unknown properties.

The device was used as a current transmitter in the range of 1 pA to 10 mA while measuring the output response of the transmitter. On this basis, the current–voltage characteristics of the presented system were determined. In the range of higher current values, the nonlinearity of the conversion characteristic is due to the presence of a rectifying diode in the feedback of the current–voltage converter. We compensated for this unwanted behavior by performing deconvolution. The characterized converter was then installed in the author’s atomic force microscope (AFM) head and was characterized on the sample. Determining the linearity interval in the characterization makes it relatively easy to convert the conversion of the output voltage of the I/U transducer into information about the value of the current flowing through the active piezoresistive lever or the resistance of the substrate. On very conductive substrates, the typical polarization value was about 10 mV. The lever force was in the range of up to 50 nN.

**Figure 9:**
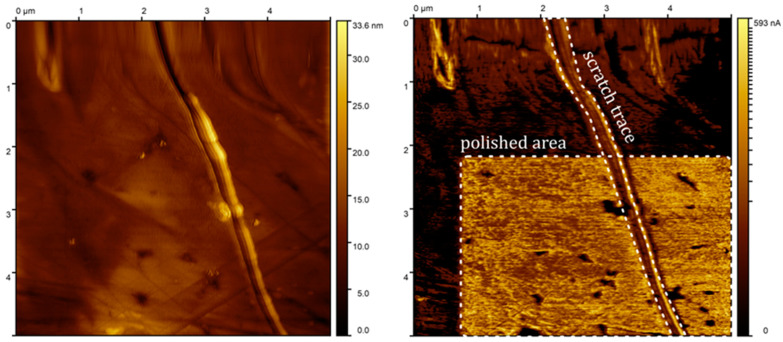
A thin copper foil sample investigated after polishing in selected area. The left image shows the topography of the scanned material, while the right image shows the current map.

Two exemplary measurements performed with the SSRM are presented. One of the samples is the technical surface of a mechanically scratched and selectively chemically polished copper alloy in polymer matrix foil (Figure 9). Imaging was conducted in a 5 × 5 μm field with a surface biased with 10 mV. The topography image presents a nanometrically flat surface with several impurities. The measurement region also contained a distinct scratch trace. The current map shows clearly that in the area that was polished, the measured current was much bigger (almost three orders of magnitude) than in the uncured area, despite no clear topographical distinction being present. Scratch trace exhibits a lower current, showing similarity between the uncured surface and bulk material.

**Figure 10:**
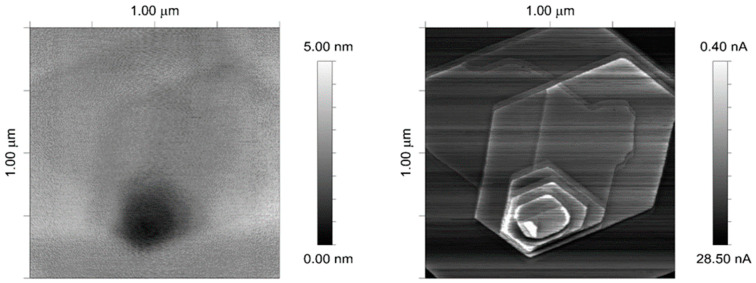
HOPG topography (**left**) and corresponding current map (**right**) with distinctively depicted graphite layers in the opening.

The second sample was an HOPG bulk crystal (Figure 10)—GRAS/1.0 type from ScanSens. In the experiment, the sample was top-layer-biased with 100 mV. Measurements of subnanoampere currents were performed. Topography imaging revealed the presence of a graphite multilayer opening, yet the exact positioning of layers was obscured by a lack of resolution. The current map showed clear distinction between layers, including elevated conductivity (greater current values) on the edges of the opening.

## 5. Discussion and Conclusions

### 5.1. Summary

In this paper, three different circuitry approaches to ultrasmall current measurements with conductive probe AFM techniques were presented. This enables measurements in both the C-AFM and SSRM modes. By applying unique solutions, we were able to overcome the limitations of typical setups and gain the measurement range of the current and the reconfiguration flexibility, which is essential in the case of correlation microscopy where various properties of the sample are investigated, as well as the data acquisition time, as the system reconfiguration time must be taken into account. The tests allowed us to confirm that the expected performance of the presented modules was obtained. Moreover, the practical verification of the usability of those solutions was presented in the case of both commercial and home-made AFM systems. 

### 5.2. Comparison with Existing Solutions

To put the solutions described above into some context, there is a need for comparison with existing commercially available solutions. This way, quick reference to the state of the art is supplied, which is especially useful for readers not familiar with the existing capabilities of measurement equipment in the field. It is worth mentioning that the comparison prepared below is not an ordinal list of equipment, but rather a list of some crucial technical parameters, as no equipment is universal but is prepared for a certain class of tasks.

Among the crucial parameters, one receives the following data: the amplification range (minimal and maximal), the bias voltage range, the measured current range, and the maximal resolution; those parameters are to be declared by the manufacturers. Values were taken from official product brochures. The different working principles of the converters make the comparison less transparent, yet it represents the general idea. The results are to be found in Table 1.

The comparison shows that the solutions presented in this paper are not much different than commercially available ones. While the working ranges are more or less comparable, the resolution is better than that of the state of the art. In conclusion, the presented solutions may be successfully used for future studies, especially ones that require superior resolution.

### 5.3. Future Applications

It should be highlighted that the effectiveness of the integration of the module with the microscope is related to the availability of the hardware interface (in particular, its parameters, such as the signal range, the conversion resolution, and the noise filtering/suppression availability). By using a microcontroller in the control unit, one can make the cooperation of those setups easy with a variety of microscopes. Following the results presented here, current measurement modules were utilized in many experiments, which are an encouraging line of development for investigation methods for measuring the nanoscale electrical properties of materials and structures, including the properties of modern materials with specially developed conductive tips [28] or modern scanning probes [29], or the properties of solar cell radial junctions. The results of the abovementioned experiments were or will be published in the near future.

## Figures and Tables

**Table 1:** Comparison of three described types of I/V converters (MFTiA, integrating, and SSRM) and three commercially available converters by JPK, Oxford Instruments, and Park Systems.

	MFTiA	Integrating I/V Converter	SSRM	TC-CAFM by JPK	ORCA Mode by Oxford Instruments	C-AFM by Park Systems
Amplification min.	10^7^	Integrating	Exponential	Hard-set	5 × 10^7^	10^3^
Amplification max.	10^10^, Exponential	Integrating	Exponential	10^9^	5 × 10^9^	10^9^
Voltage range	±6 V	±12 V	±12 V	±10 V	±10 V	±10 V
Current range	±100 μA	±688.12 nA	100 fA–10 μA	±10 nA	−6 pA–+10 μA	±10 mA
Resolution	<3 pA	<2.3 fA	<100 fA	100 fA	<1 pA	<300 fA

## Data Availability

The experimental data can be provided by the authors on demand.
